# Antiviral susceptibility of clade 2.3.4.4b highly pathogenic avian influenza A(H5N1) viruses from humans in the United States, October 2024 to February 2025

**DOI:** 10.1080/22221751.2025.2601372

**Published:** 2025-12-15

**Authors:** Philippe Noriel Q. Pascua, Anton P. Chesnokov, Ha T. Nguyen, Chloe Champion, Rongyuan Gao, Juan A. De La Cruz, Yunho Jang, Yasuko Hatta, Zhu Guo, Timothy M. Uyeki, Han Di, James Stevens, Charles T. Davis, Larisa V. Gubareva

**Affiliations:** Influenza Division, Centers for Disease Control and Prevention, Atlanta, GA, USA

**Keywords:** A(H5N1) viruses, neuraminidase, neuraminidase inhibitors, bovine flu, drug resistance, baloxavir

## Abstract

Since October 2024, 55 human cases of influenza A(H5N1), clade 2.3.4.4b, were reported in the US. Sequencing of 46 viruses identified genotypes B3.13, D1.1, and D1.3. Virus genomes were analyzed for mutations associated with reduced antiviral susceptibility. Except for two, viruses from human cases were assessed as susceptible to antivirals and confirmed by *in vitro* susceptibility testing of representative viruses with M2 blockers, neuraminidase (NA) inhibitors, and the polymerase acidic (PA) inhibitor baloxavir. One D1.1 virus had an M2-S31N substitution, which conferred cross-resistance to M2 blockers. One B3.13 virus had a PA-I38M substitution and displayed 17-fold reduced baloxavir susceptibility in cell culture. In mice, baloxavir, given orally at 5, 15, and 45 mg/kg *bid* for 5 days starting at 2 h post-infection, was notably less effective against the PA-I38M virus compared to the closely matched control virus, as shown by survival rate, time-to-death, weight loss, and viral replication in various organs. Replication of the PA-I38M virus was mildly attenuated in mice but not in cell culture. Viruses collected from animals also contained mutations associated with reduced antiviral susceptibility, including M2-S31N, PA-I38 T/M, and NA-H275Y, albeit at low frequency. Regardless of genotype, most mutations were found in NA. Using recombinant NA proteins generated from B3.13 and D1.1 backgrounds, we showed that among mutations which reduced inhibition by NA inhibitors, only NA-H275Y had no effect on enzyme function. Spontaneous emergence of drug-resistant influenza viruses, especially those that maintain replicative fitness, pose persistent threat to human health and must be closely monitored.

## Introduction

Since late 2021, clade 2.3.4.4b highly pathogenic avian influenza (HPAI) A(H5N1) viruses have caused widespread outbreaks in wild birds and poultry in North America. These viruses have reassorted with low pathogenicity avian influenza A viruses that led to co-circulation of genetically diverse HPAI A(H5) viruses (https://github.com/USDA-VS/GenoFLU) [1]. To date, over 100 distinct genotypes of clade 2.3.4.4b viruses have been identified in North American birds and other animals. In early 2024, a genotype B3.13 virus spilled over to dairy cows in the United States (U.S.) causing unprecedented multi-state outbreaks [2–4]. The interspecies transmission of clade 2.3.4.4b viruses also led to sporadic human cases, mostly among individuals working closely with infected cows or poultry. Thus, the ongoing clade 2.3.4.4b virus outbreaks in animals are a concern to public health.

Between 28 March 2024, and 12 February 2025, a total of 70 human cases of A(H5N1) in the U.S. were confirmed by the Centers for Disease Control and Prevention (CDC) [5,6]. The only other confirmed case occurred in 2022 [7]. Forty-one of the A(H5N1) cases were exposed to sick cows and 26 worked with infected poultry; the source of exposure in three cases was not determined [6]. While most of the human infections were relatively mild, four were hospitalized, including three with pneumonia, and one of the hospitalized patients died.

Antiviral medications play an important role in the treatment of novel influenza A virus infections, especially for viruses associated with severe disease in infected humans, for which vaccines are not available. Three classes of direct-acting antivirals are approved by the Food and Drug Administration (FDA) for treatment of influenza in the U.S. (https://www.cdc.gov/flu/treatment/antiviralresistance.html). The oldest antivirals are M2 blockers (amantadine and rimantadine), which target the M2 ion channel protein [8]. The neuraminidase (NA) inhibitor oseltamivir is the most widely used influenza antiviral [9], and like M2 blockers, it is administered orally in multiple doses. Other NA inhibitors are the inhaled zanamivir and the intravenously delivered peramivir. An additional inhaled NA inhibitor, laninamivir, is approved in Japan but not the U.S. [10]. Baloxavir (marboxil) is an inhibitor of the cap-dependent endonuclease activity of the polymerase acidic (PA) protein that is prescribed as a single pill. CDC recommends oseltamivir for treatment and post-exposure prophylaxis of HPAI A(H5N1) virus infection [11–13]. For hospitalized patients with severe infection, combination antiviral treatment (e.g. oseltamivir and baloxavir) can be considered [11]; triple combination antiviral treatment with oseltamivir, baloxavir, and amantadine was administered to a critically ill A(H5N1) patient in Canada [14].

The effectiveness of antivirals can be compromised by mutations in the viral genome. M2 blockers are not recommended by CDC for treatment of infection with seasonal influenza A viruses as they acquired the resistance-conferring amino acid substitution S31N in the M2 protein [15]. During the 2008–2009 influenza season, the high prevalence of oseltamivir resistance among pre-2009 pandemic seasonal influenza A(H1N1) viruses affected clinical practice by presenting challenges for selecting antiviral medications [16]. These viruses contained NA-H275Y, which is associated with resistance to oseltamivir and peramivir, but does not alter susceptibility to zanamivir [17]. To date, widespread resistance to baloxavir among seasonal influenza viruses has not been identified, but PA-I38 T has emerged as the principal pathway to resistance. Besides the most common mutations, there are numerous others that have been shown or are suspected of reducing susceptibility to approved influenza antivirals.

Mutations that reduce susceptibility to antivirals can emerge spontaneously, by gene reassortment, or following exposure to antivirals. A small number of clade 2.3.4.4b A(H5N1) viruses collected from wild birds and mammals in the U.S. during the ongoing outbreak have been reported to contain mutations that may reduce the effectiveness of one or more approved antivirals [18,19]. Such mutations were also found in viruses collected from poultry and dairy cows (e.g. NA-T438I). A recent report from Canada raised concerns about the local transmission of oseltamivir-resistant clade 2.3.4.4b viruses containing NA-H275Y among poultry at several commercial poultry farms [20].

In the report by Rofles et al. [6], a brief description of mutations known to confer antiviral resistance was provided for all confirmed human cases in the U.S. In our previous study, we provided a detailed analysis of the antiviral susceptibility of viruses collected prior to October 2024 [21]. Here, we extended this analysis to include viruses from human cases reported since October 2024. This study encompasses viruses from A(H5N1) case-patients who were working at dairy and poultry farms, as well as viruses collected from animals during the same period. In addition to the previously used methodology, we applied recombinant NA proteins to assess how various identified NA mutations affect susceptibility to NA inhibitors. Furthermore, for the first time, using a mouse model we evaluated the therapeutic effect of baloxavir treatment against a HPAI A(H5N1) virus that displays reduced baloxavir susceptibility *in vitro*.

## Materials and methods

### Viruses

Clinical specimens (conjunctival and respiratory) that tested positive for HPAI A(H5N1) viruses were submitted by U.S. public health laboratories to CDC, Atlanta, GA (Table S1). The A(H5N1) reference virus, A/bald eagle/Florida/22-006544-004/2022 (eagle/FL/22), was provided by the U.S. Department of Agriculture [18]. Viruses were propagated in 10-day old embryonated chicken eggs or in Madin-Darby canine kidney (MDCK) cells (ATCC). The CDC Antiviral Susceptibility Reference Virus Panels (FR-1755 ver3 and FR-1678 ver1.1; International Reagent Resource) were used as controls in phenotypic assays. Seasonal A(H1N1)pdm09 viruses with or without M2-S31N from the CDC repository were included for reference purposes. Handling and experiments with HPAI A(H5N1) viruses were conducted in an enhanced biosafety level 3 containment facility.

### Next generation sequencing (NGS) analysis

HPAI A(H5N1) virus genome sequences were generated using NGS (Illumina), analyzed by the iterative refinement meta-assembler (IRMA) [22] with single-nucleotide variant threshold of 5%. All sequences were deposited in the shared database Global Initiative on Sharing All Influenza Data (GISAID) (Table S1).

### Virus growth experiments

MDCK, MDCK-SIAT1 (MilliporeSigma), and humanized MDCK (hCK; kindly provided by Dr. Yoshihiro Kawaoka) cell monolayers in 6-well plates were inoculated at a multiplicity of infection of 0.00005 (50 EID_50_; 50 TCID_50_-MDCK-SIAT1), followed by adsorption (30 min) and incubation at 37°C in 5% CO_2_; medium supplemented with 2 µg/mL TPCK-treated trypsin was used for A(H3N2) viruses only. Supernatants were collected and 50% tissue culture infectious dose (TCID_50_) titres were determined using MDCK-SIAT1 cells. Titres of virus pairs were compared by Sidak’s two-way ANOVA (Prism 10, GraphPad).

### Antiviral compounds

Stocks of M2-blockers, amantadine hydrochloride (MilliporeSigma) and rimantadine (Hoffman-La Roche), and NA-inhibitors, oseltamivir carboxylate (oseltamivir), zanamivir, peramivir, and laninamivir (BioSynth), were dissolved in distilled water. The active metabolite PA-inhibitor baloxavir acid (MedChemExpress) was dissolved in DMSO. Antiviral stocks (stored at −20°C) were serially diluted in respective assay buffer/medium. Working concentrations of the prodrug PA-inhibitor baloxavir marboxil (baloxavir; MedChemExpress) for mice treatment were prepared in 0.5% methylcellulose and stored at 4°C.

### Generation and quantification of recombinant NA

Recombinant neuraminidases (recNAs) were generated as previously described [23]. Briefly, cDNA encoding residues 82–469 of the NA genes of A/California/148/2024 (H5N1) and A/Washington/239/2024 (H5N1) were synthesized (GenScript) and subcloned into a pIEx-4 vector (MilliporeSigma). Constructs were transiently transfected into Sf9 cells (EMD Millipore) and secreted recNA were quantified by Western blot. recNA were assessed for functional activity, using solid-phase fetuin [24] and/or 2′-(4-methylumbelliferyl)-α-D-N-acetylneuraminic acid (MUNANA) and analyzed by the NI assay described below.

### Neuraminidase inhibition (NI) assay

The NA-Fluor™ Influenza Neuraminidase Assay Kit (Applied Biosystems) was used to test viruses and recNAs with NA inhibitors in 96-well black microplates as previously described [25]: normalized viruses/recNA were incubated with MUNANA substrate for 1 h in the presence of NA inhibitors and fluorescence was measured using Cytation 7 (Agilent-Biotek). Fold-rise in 50% inhibitory concentration (IC_50_) of viruses and recNA was compared to the median for the respective genotype (test viruses), wildtype virus (control viruses), or recNA proteins. Fold rise was interpreted as normal inhibition if <10-fold, reduced inhibition (RI) if 10- to 100-fold, or highly-reduced inhibition (HRI) if >100-fold [26].

### Influenza replication inhibition neuraminidase-based assay (IRINA)

Susceptibility to M2 blockers and baloxavir acid was assessed using the cell culture-based, single-cycle replication assay IRINA as previously described [21,27]. Briefly, in a 96-well microplate, normalized virus, serially diluted antiviral, and MDCK-SIAT1 cell suspension were incubated at 37°C for 7 h, after which supernatant was removed and enzyme activity of NAs expressed on cell surface was measured using MUNANA. The working range for fluorescence was equivalent to 200–2000 pmol of 4-methylumbelliferone.

### In vivo baloxavir treatment in mice

Specific pathogen-free, female, 6-week-old BALB/c mice (Jackson Laboratory) were acclimated for 3 days prior to virus inoculation. The 50% mouse lethal dose (MLD_50_, n = 8) was determined by intranasal inoculation with 10-fold serially diluted virus in PBS (10^0.1^–10^3^ EID_50_/TCID_50_ per 50 µL). Daily weights and signs of disease were recorded. End-point criteria were 25% weight loss, neurological signs, or combination of 15–25% weight loss and signs of disease.

Animals (n = 16) were inoculated with 5× MLD_50_ (30 TCID_50_/50 µL) of HPAI A(H5N1) viruses (A/California/147/2024 or A/California/150/2024) and treated by oral gavage with baloxavir (marboxil) twice daily (*bid*, 12 h apart) at 0, 5, 15, or 45 mg/kg/dose, 100 µL per mouse, starting at 2 h post-infection for 5 days (10 total treatments). Mice were observed daily. The dose setting was based on the pharmacokinetics profile of baloxavir in mice where oral administration of 15 mg/kg baloxavir *bid* for 5 days is considered clinically equivalent to the human dose [28,29]. The three times lower and higher doses were included to examine dose-dependency.

Animal organs were collected at 3 and 5 days post-infection (dpi; 4 mice per time point) and homogenized (Omni Bead Ruptor, Revvity) in 1 mL of DMEM (Gibco) containing penicillin/streptomycin (Gibco). Cellular debris was cleared by centrifugation at 2000 × g for 10 min. Supernatants were used to determine infectious virus titre (TCID_50_ per g) in MDCK-SIAT1 cells using the Spearman-Kärber method.

### Ethics statement

Animal experiments were conducted following a protocol approved by the CDC Institutional Animal Care and Use Committee (IACUC) and in strict compliance with CDC IACUC guidelines, which adhere to PHS Policy, the Animal Welfare Act (U.S. Department of Agriculture), and the Guide for Care and Use of Laboratory Animals. All procedures were performed under enhanced animal biosafety level 3 conditions.

### Statistical analysis

Statistical analyses were performed using GraphPad Prism 10.0 (GraphPad Software, Inc.). All graphs were generated in GraphPad Prism. Survival graphs were generated by the Kaplan-Meier survival curves and data were compared using the log-rank Mantel–Cox test. Two-way ANOVA with Sidak’s or Tukey’s multiple comparison *post-hoc* test was used for statistical comparison of viral titres from *in vitro* growth experiments and in mice tissues.

## Results

From October 2024 to February 2025, there were 55 confirmed human cases associated with clade 2.3.4.4b A(H5N1) viruses in nine U.S. states; most were detected in California (n = 37) and Washington (n = 11) (Table S1). Viruses from human cases in California belonged to genotype B3.13, while others belonged to D1.1, except for one D1.3 virus (Table S1). Notably, the three gene segments (M, NA, and PA) that encode antiviral-targeted proteins were derived from various avian influenza A virus lineages (Table S2). All human viruses analyzed in this study were collected prior to antiviral treatment [6]. Sequences of animal viruses collected from 1 October 2024 to 28 February 2025 that were shared with CDC or submitted to GISAID by 23 June 2025 were also examined.

### Susceptibility to M2 blockers

The M2 protein sequences of viruses from humans were highly similar to that of eagle/FL/22 (genotype B1.1) (Tables S3 and S4), whose susceptibility to M2 blockers was previously demonstrated [21]. Only one virus, A/Iowa/124/2024 (genotype D1.1), had a molecular marker of M2 blocker-resistance (M2-S31N). Its drug-resistant phenotype was confirmed in cell culture using IRINA, where replication was not inhibited even at 1 µg/mL (Table 1). Other D1.1 and B3.13 viruses displayed normal susceptibility, with rimantadine 50% effective concentrations (EC_50_s) being somewhat lower (3.14–6.48 ng/mL) compared to amantadine (9.25–21.54 ng/mL).

**Table 1.** Susceptibility of clade 2.3.4.4b HPAI A(H5N1) viruses to M2 blockers by IRINA.

Influenza A(H5N1) virus	EC_50_ ± SD, ng/mL^a^	
	Amantadine	Rimantadine
Genotype B3.13		
A/California/135/2024	18.27 ± 1.09	4.83 ± 0.15
A/California/146/2024	21.54 ± 3.86	6.48 ± 1.14
Genotype D1.1		
A/Washington/239/2024	9.25 ± 2.47	3.14 ± 0.31
A/Washington/240/2024	11.41 ± 3.00	3.46 ± 0.08
A/Iowa/24/2024, **M2-S31N**	**>1000**	**>1000**
Control viruses		
A/bald eagle/FL/2022 (H5N1)^b^	20.51 ± 4.96	5.74 ± 1.31
A/Wisconsin/53/2009 (H1N1)pdm09	25.93 ± 1.51	6.08 ± 0.90
A/California/07/2009 (H1N1)pdm09, **M2-S31N**	**>1000**	**>1000**

HPAI, highly pathogenic avian influenza; IRINA, Influenza replication inhibition neuraminidase-based assay.

^a^ Data shown are mean and standard deviation (SD) of at least three experiments. Concentration range of the M2 blockers was 3.9–1000 ng/mL.

^b^ Clade 2.3.4.4b A(H5N1) virus, A/bald eagle/Florida/22-006544-004/2022 (submitted as A/bald eagle/Florida/W22-134-OP/2022; GISAID ID: EPI_ISL_15063846, genotype B1.1) was used as a drug-sensitive A(H5N1) control virus.

Sequence analysis of A(H5N1) viruses collected from animals in the US during the same period showed that of 5,536 viruses, 63 (1.3%) had M2-S31N (all collected from birds), while fewer viruses had other markers: L26F (n = 1), V27A (n = 10), and A30 T (n = 4) (Table S5). Interestingly, a cluster of five viruses from chickens sharing an identical genome, including M2-S31N, were collected in the same locality in Iowa 4–5 days before detection of the human case, suggesting potential spillover (Table S6).

### Susceptibility to NA inhibitors

Compared to eagle/FL/22, genotype B3.13 viruses had only a few amino acid substitutions in NA, whereas genotype D1.1 and D1.3 viruses differed by 14–16 and 9 substitutions, respectively (Tables S3 and S4). None of these viruses contained NA-H275Y or any other substitutions known to reduce inhibition by NA inhibitors. Nevertheless, we flagged 11 viruses from human A(H5N1) cases whose NA sequences contained substitutions of interest at the following residues: V116I and S439G in B3.13 genotype and S247N and K432R in D1.1.

A set of virus isolates (n = 17) from B3.13 and D1.1 genotypes was tested with the NI assay with four NA inhibitors. These viruses were deemed susceptible based on IC_50_ values, which fell into sub-nanomolar to low nanomolar ranges (Table 2). NA-K432R had no effect on the inhibition profile (1-fold), while NA-S439G conferred an 8-fold rise in zanamivir IC_50_ (normal inhibition). The effect of NA-V116I or NA-S247N was not assessed because their respective viruses were not recovered.

**Table 2.** Susceptibility of clade 2.3.4.4b HPAI A(H5N1) viruses to NA and PA inhibitors.

Influenza A virus	NA inhibitor IC_50_ ± SD, nM (fold-change)^a^				PA inhibitor EC_50_ ± SD, nM (fold-change)^b^
	Oseltamivir	Zanamivir	Peramivir	Laninamivir	Baloxavir
Genotype B3.13, median IC_50_ (n = 10)	3.50	0.20	0.07	0.17	0.50
A/California/135/2024, NA-S439G	6.88 ± 0.91 (2)	1.26 ± 0.37 (6)	0.16 ± 0.01 (2)	0.20 ± 0.02 (1)	0.99 ± 0.13 (2)
A/California/146/2024, NA-S439G	7.43 ± 0.69 (2)	1.53 ± 0.13 (8)	0.19 ± 0.01 (3)	0.23 ± 0.02 (1)	0.72 ± 0.07 (1)
A/California/147/2024	3.62 ± 0.63 (1)	0.18 ± 0.03 (1)	0.07 ± 0.01 (1)	0.15 ± 0.01 (1)	0.40 ± 0.11 (1)
A/California/148/2024	3.31 ± 0.39 (1)	0.17 ± 0.00 (1)	0.07 ± 0.01 (1)	0.15 ± 0.01 (1)	0.26 ± 0.13 (0.4)
A/California/150/2024, **PA-I38M**	2.77 ± 0.37 (1)	0.19 ± 0.03 (1)	0.07 ± 0.01 (1)	0.13 ± 0.01 (1)	5.42 ± 1.71 (**11; 17**)^c^
A/California/151/2024	3.54 ± 0.39 (1)	0.21 ± 0.06 (1)	0.07 ± 0.01 (1)	0.16 ± 0.02 (1)	0.72 ± 0.18 (1)
A/California/152/2024	3.45 ± 0.23 (1)	0.20 ± 0.02 (1)	0.08 ± 0.01 (1)	0.17 ± 0.01 (1)	0.45 ± 0.18 (1)
A/California/153/2024	3.56 ± 0.82 (1)	0.18 ± 0.02 (1)	0.06 ± 0.02 (1)	0.17 ± 0.01 (1)	0.59 ± 0.20 (1)
A/California/155/2024	2.71 ± 0.51 (1)	0.16 ± 0.01 (1)	0.08 ± 0.01 (1)	0.19 ± 0.03 (1)	0.41 ± 0.08 (1)
A/California/173/2024	3.26 ± 0.32 (1)	0.23 ± 0.02 (1)	0.06 ± 0.01 (1)	0.15 ± 0.01 (1)	0.69 ± 0.16 (1)
Genotype D1.1, median IC_50_ (n = 7)	2.87	0.36	0.07	0.13	0.34
A/Iowa/24/2024	2.45 ± 0.25 (1)	0.34 ± 0.04 (1)	0.07 ± 0.01 (1)	0.14 ± 0.01 (1)	0.26 ± 0.06 (1)
A/Nevada/10/2025	2.45 ± 0.19 (1)	0.36 ± 0.06 (1)	0.07 ± 0.01 (1)	0.13 ± 0.01 (1)	0.34 ± 0.17 (1)
A/Washington/239/2024, NA-K432R	3.05 ± 0.61 (1)	0.28 ± 0.04 (1)	0.08 ± 0.01 (1)	0.14 ± 0.02 (1)	0.35 ± 0.13 (1)
A/Washington/240/2024, NA-K432R	3.01 ± 0.55 (1)	0.30 ± 0.06 (1)	0.07 ± 0.01 (1)	0.14 ± 0.01 (1)	0.34 ± 0.06 (1)
A/Washington/254/2024, NA-K432R	2.87 ± 0.28 (1)	0.39 ± 0.06 (1)	0.06 ± 0.00 (1)	0.11 ± 0.01 (1)	0.43 ± 0.05 (1)
A/Washington/255/2024, NA-K432R	2.73 ± 0.08 (1)	0.43 ± 0.08 (1)	0.07 ± 0.01 (1)	0.13 ± 0.01 (1)	0.27 ± 0.07 (1)
A/Wyoming/01/2025	2.94 ± 0.06 (1)	0.46 ± 0.04 (1)	0.07 ± 0.01 (1)	0.11 ± 0.02 (1)	0.63 ± 0.28 (2)
Control viruses	** **				
A/bald eagle/FL/2022 (H5N1)	3.09 ± 0.62	0.22 ± 0.04	0.08 ± 0.02	0.17 ± 0.02	0.39 ± 0.16
A/Illinois/45/2019 (H1N1)pdm09	0.28 ± 0.04	0.15 ± 0.02	0.04 ± 0.01	0.17 ± 0.02	0.84 ± 0.18
A/Alabama/03/2020 A(H1N1)pdm09, **NA-H275Y**	169.33 ± 27.14 (**605**)	0.23 ± 0.04 (2)	17.65 ± 2.05 (**441**)	0.35 ± 0.03 (2)	0.67 ± 0.28
A/Illinois/08/2018 (H1N1)pdm09	0.34 ± 0.03	0.21 ± 0.04	0.04 ± 0.01	0.15 ± 0.02	0.96 ± 0.37
A/Illinois/08/2018 (H1N1)pdm09, **PA-I38T**	0.34 ± 0.05	0.19 ± 0.05	0.04 ± 0.01	0.12 ± 0.03	70.81 ± 12.45 (**74**)
A/Louisiana/50/2017 (H3N2)	0.10 ± 0.02	0.21 ± 0.02	0.07 ± 0.01	0.32 ± 0.06	0.80 ± 0.28
A/Louisiana/49/2017 (H3N2), **PA-I38M**	0.12 ± 0.02	0.21 ± 0.04	0.06 ± 0.00	0.32 ± 0.03	9.95 ± 2.71 **(12)**

Data shown are mean and standard deviation (SD) of at least three experiments. HPAI, highly pathogenic avian influenza; NA, neuraminidase; PA, polymerase acidic.

^a^ Inhibition of NA enzyme activity was assessed in a fluorescent NI assay. Fold-change in **bold** indicate reduced inhibition (RI, >10-fold) or highly reduced inhibition (HRI, >100-fold) by NA inhibitor relative to corresponding wildtype virus.

^b^ Susceptibility to baloxavir acid, the active form of baloxavir marboxil, was assessed using the cell culture-based assay IRINA at 7 hpi. Fold-change in **bold** indicate baloxavir EC_50_s > 3-fold relative to PA sequence-matched wildtype virus EC_50_.

^c^ Fold-change in **bold** indicate baloxavir EC_50_s > 3-fold relative to genotype-specific median EC_50_ (**11**-fold) or the PA sequence-matched wildtype virus (A/California/147/2024; **17**-fold) EC_50_.

NA sequence analysis was extended to HPAI A(H5N1) viruses collected from animals, and of 5,382 sequences, three (0.06%) D1.1 viruses collected from wild birds in the U.S. in December had NA-H275Y. We also flagged substitutions at 23 other residues (Table S5). The most common NA substitutions (>10 viruses) were: V116I (n = 124, 2.30%), V116A (n = 19, 0.35%) Q136H (n = 17, 0.32%); I427 T (n = 12, 0.22%); K432R (n = 43, 0.80%), T438A (n = 11, 0.20%), and S439G (n = 22, 0.41%). Of note, T438I was present at only 0.15%, which is much lower than the 3.6% reported during 2022–2023 [19]. Interestingly, the new mutation T438A was only seen in viruses from dairy cows.

To evaluate the effect of NA-H275Y and eight other flagged substitutions, we generated recNA proteins using NAs from B3.13 and D1.1 genotype viruses. As expected, in the NI assay, NA-H275Y conferred HRI by oseltamivir (159- to 184-fold) and peramivir (1064- to 1724-fold) but normal inhibition by zanamivir and laninamivir (Table 3). NA-V116I had no effect, whereas NA-V116A conferred RI by zanamivir (14- to 22-fold) in both backgrounds and RI by peramivir (12-fold) in D1.1 only. Although slightly elevated IC_50_s were observed for NA-I223T and NA-S247N, results were interpreted as normal inhibition by all antivirals. NA-I427T and NA-T438I conferred similar inhibition profiles in both backgrounds: RI/HRI by zanamivir, peramivir, and laninamivir, but normal inhibition by oseltamivir. The rarely found NA-E119D and NA-R152G had the most pronounced effect: HRI by zanamivir, peramivir, and laninamivir, and RI by oseltamivir (Table 3).

**Table 3.** Effects of substitutions in the clade 2.3.4.4b HPAI A(H5N1) recombinant NA proteins on enzyme activity and its inhibition by antivirals.

NA mutation	N2 numbering	IC_50_± SD (fold-change)^a^				Relative NA activity (%)^b^
		Oseltamivir	Zanamivir	Peramivir	Laninamivir	
A/California/148/2024 (genotype B3.13)						
WT	–	3.99 ± 1.03 (1)	0.24 ± 0.06 (1)	0.08 ± 0.01 (1)	0.16 ± 0.02 (1)	100
V116I	116	4.64 ± 1.71 (1)	0.42 ± 0.15 (2)	0.09 ± 0.02 (1)	0.15 ± 0.04 (1)	132
V116A	116	12.42 ± 2.92 (3)	3.35 ± 0.90 (**14**)	0.46 ± 0.04 (6)	0.42 ± 0.04 (3)	67
E119D	119	73.35 ± 7.66 (**18**)	1507.52 ± 118.60 (**6281**)	156.57 ± 10.35 (**1957**)	238.11 ± 20.33 (**1488**)	6
R152G	152	64.96 ± 5.85 (**16**)	305.52 ± 17.40 (**1273**)	64.79 ± 1.54 (**810**)	110.55 ± 16.05 (**691**)	5
I223T	222	13.49 ± 1.42 (3)	0.60 ± 0.12 (3)	0.27 ± 0.01 (3)	0.27 ± 0.04 (2)	156
S247N	246	26.25 ± 4.23 (7)	0.48 ± 0.14 (2)	0.30 ± 0.06 (4)	0.36 ± 0.11 (2)	93
H275Y	274	634.08 ± 76.80 (**159**)	0.37 ± 0.06 (2)	85.11 ± 22.52 (**1064**)	0.54 ± 0.15 (3)	100
I427T	427	29.09 ± 5.80 (7)	18.11 ± 2.46 (**75**)	1.27 ± 0.52 (**16**)	2.34 ± 0.81 (**15**)	27
T438I	439	28.23 ± 3.22 (7)	28.61 ± 5.62 (**119**)	1.59 ± 0.50 (**20**)	3.89 ± 1.52 (**24**)	10
A/Washington/239/2024 (genotype D1.1)						
WT	–	3.52 ± 0.96 (1)	0.31 ± 0.07 (1)	0.06 ± 0.00 (1)	0.14 ± 0.02 (1)	100
V116I	116	4.41 ± 1.78 (1)	0.64 ± 0.28 (2)	0.08 ± 0.01 (1)	0.15 ± 0.01 (1)	138
V116A	116	9.82 ± 3.69 (3)	6.83 ± 0.55 (**22**)	0.73 ± 0.23 (**12**)	0.78 ± 0.12 (6)	52
E119D	119	76.11 ± 16.83 (**22**)	2502.31 ± 192.35 (**8072**)	192.79 ± 32.58 (**3213**)	382.87 ± 40.38 (**2735**)	4
R152G	152	47.51 ± 14.17 (**13**)	543.72 ± 44.18 (**1754**)	90.89 ± 19.52 (**1515**)	200.56 ± 57.30(**1433**)	4
I223T	222	11.96 ± 1.79 (3)	1.13 ± 0.06 (4)	0.24 ± 0.03 (4)	0.23 ± 0.00 (2)	130
S247N	246	17.87 ± 2.61 (5)	0.54 ± 0.04 (2)	0.22 ± 0.03 (4)	0.24 ± 0.03 (2)	96
H275Y	274	648.16 ± 126.97 (**184**)	0.38 ± 0.09 (1)	103.43 ± 2.33 (**1724**)	0.43 ± 0.08 (3)	98
I427T	427	21.80 ± 4.74 (6)	27.94 ± 7.68 (**90**)	1.38 ± 0.14 (**23**)	3.45 ± 0.33 (**25**)	17
T438I	439	10.43 ± 2.86 (3)	33.36 ± 4.04 (**108**)	1.54 ± 0.23 (**26**)	4.61 ± 0.92 (**33**)	14

Recombinant neuraminidase (recNA) proteins were generated in the NA genetic background of A/California/148/2024 or A/Washington/239/2024 virus. HPAI, highly pathogenic avian influenza.

^a^ Inhibition of enzyme activity of recNA proteins was determined in NI assay; fold-change in **bold** indicate RI (>10-fold) or HRI (>100-fold) by NA inhibitors relative to corresponding wild type recNA. Data shown are mean and standard deviation (SD) of three results from two independent experiments.

^b^ NA activity was measured using small substrate MUNANA; the relative NA activities of the wildtype recNAs are similar (36,360 vs 38,110 RFU/ng).

Next, we assessed whether the above NA substitutions also affect enzyme activity. We utilized the substrate MUNANA to measure NA activity of the recNAs in the panel. Wildtype recNAs from both backgrounds had similar enzyme activities and the effects, if any, from the substitutions were the same in both backgrounds (Table 3). The three substitutions that did not affect antiviral inhibition either increased NA activity (NA-V116I and NA-I223T) or only slightly reduced it (NA-S247N). NA-V116A reduced NA activity by 33–58%, whereas NA-E119D, NA-R152G, NA-I427T, and NA-T438I had greater negative effects (73–96%).

Although NA-H275Y was able to confer HRI by oseltamivir and zanamivir, there was no effect on enzyme activity in either genetic background. This observation was reinforced when testing was done using the natural substrate fetuin (Table S7).

### Antiviral susceptibility to baloxavir

PA protein sequences of genotype B3.13 differ from D1.1 and D1.3 by numerous amino acids (Tables S3 and S4). One B3.13 virus from a human A(H5N1) case, A/California/150/2024 (CA/150), had PA-I38M, a molecular marker associated with reduced baloxavir susceptibility.

A set of viruses were tested in cell culture with baloxavir (Table 1). Except for CA/150, all viruses were deemed susceptible based on sub-nanomolar EC_50_s. PA-I38M in CA/150 background conferred 11- and 17-fold rise in EC_50_ compared to the genotype-specific median and PA sequence-matched virus A/California/147/2024 (CA/147), respectively. In the same assay, the seasonal virus with PA-I38M showed 10-fold rise over its sequence-matched control.

Analysis of 5,090 PA sequences of animal viruses revealed 55 (1.08%) with the following substitutions of interest: E18G/K, A36S/T/V, A37S, I38M/T/V, and V122A (Table S5). PA-I38M was present in seven viruses from both birds and mammals in different states. PA-I38T is considered the principal marker of baloxavir resistance and was found in viruses collected from birds and mammals. Interestingly, of the 13 viruses with PA-I38T, seven were collected from chickens at the same locality and on the same date.

### In vitro replicative fitness of viruses with and without PA-I38M

As part of risk assessment, we evaluated whether PA-I38M attenuates replication of the human A(H5N1) virus in cell culture. The replication kinetics of CA/150 PA-I38M and wildtype CA/147 were determined in three cell lines: MDCK, MDCK-SIAT1, and hCK. For reference purposes, we used a seasonal A(H3N2) virus pair with and without PA-I38M, which replicated similarly in each cell line (Figure S1A, S1C, S1E). Compared to these seasonal viruses, both A(H5N1) viruses replicated to higher titres faster, peaking at 48 h in all cell lines (Figure S1B, S1D, S1F). As seen with the A(H3N2) virus, PA-I38M did not attenuate replication of CA/150. In hCK cells, this mutant grew to slightly higher titres (Figure S1F), however, this does not mean that PA-I38M offered a growth advantage, as this mutant has three additional differences in the genome (PB2-T58A, PB1-T6I, and NA-S369N) compared to CA/147.

### Baloxavir treatment of mice infected with A(H5N1) viruses with and without PA-I38M

The mouse model was used to assess whether PA-I38M in the A(H5N1) virus (*i*) attenuates growth *in vivo*, and (*ii*) reduces the therapeutic efficacy of baloxavir. The MLD_50_ of CA/147 and CA/150 PA-I38M viruses were similar (10^0.75^, ∼6 TCID_50_). However, mean time-to-death (TTD) of mice infected with CA/150 PA-I38M was delayed by 1–2 days (Figure S2), suggesting a mild attenuation.

**Figure 1.**
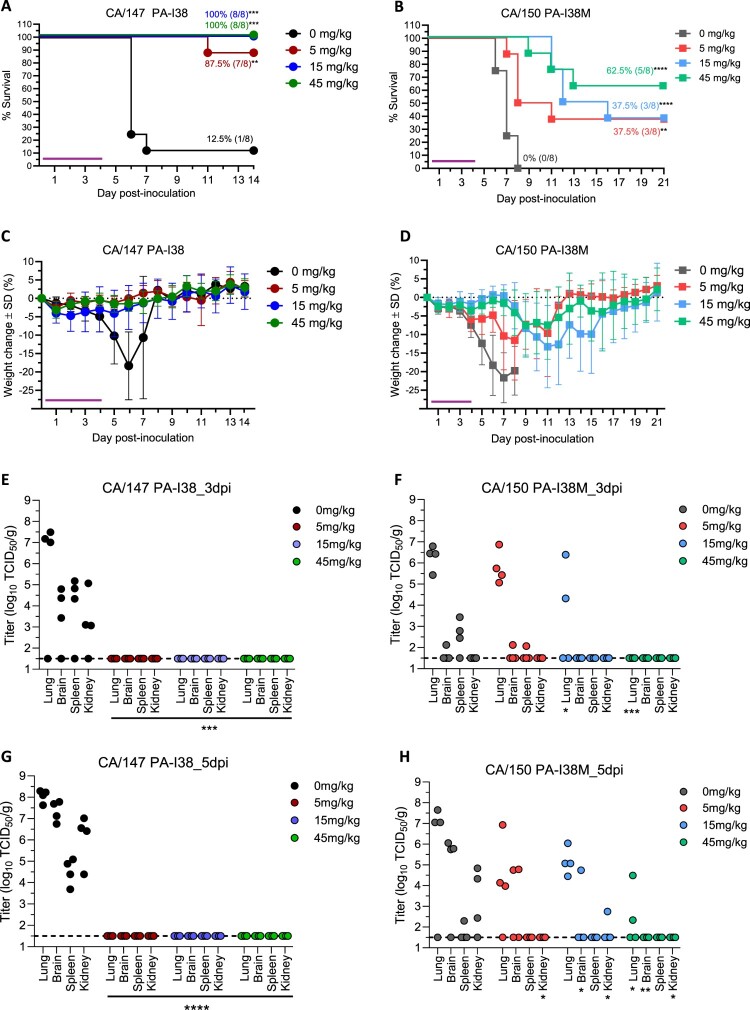
Effect of baloxavir treatment in mice inoculated with A(H5N1) virus with or without PA-I38M. Female six-week-old BALB/c mice (n = 16/group) were inoculated with 5× MLD_50_ of A/California/147/2024 (CA/147, wildtype) (A,C,E,G) or A/California/150/2024 (CA/150, PA-I38M mutation) (B, D, F, H). Immediately after infection (∼2hpi), all inoculated mice we treated twice daily by oral gavage with baloxavir or placebo for 5 days (purple horizontal bar). Survival (A, B) and body weights (C, D) were monitored daily for 14 (CA/147, A and C) or 21 (CA/150, B and D) days (n = 8 mice per treatment dose). Data are mean percentages ± standard deviation (S.D.) of the starting weight. Survival curves were compared by the log-rank Mantel-Cox test. Virus titres in the lungs, brain, spleen and kidney were determined by performing TCID_50_ in MDCK-SIAT1 cells at day 3 (E, F) and day 5 (G, H) post-infection (n = 4 mice per treatment condition). Data are means ± S.D.; points represent data from individual mice. The lower limit of detection is indicated by the horizontal dashed line (1.50 log_10_ TCID_50_/g). TCID_50_/g, 50% tissue infectious dose per gram of tissue. Two-way ANOVA with Tukey’s multiple comparison test was used for statistical comparison of tissue titres from treated and untreated groups (*p<0.05; **p < 0.01; ***p < 0.001; ****p < 0.0001).

Next, mice were inoculated with 5× MLD_50_ to achieve near 100% lethality. At 2 hpi, animals were treated by oral gavage with baloxavir at 0, 5, 15, or 45 mg/kg *bid* for 5 days. Mock-treated mice infected with CA/147 gradually lost weight and succumbed to infection by 7 dpi (12.5% survival; 6-day mean TTD) (Figure 1(A and C)). All mock-treated mice infected with CA/150 PA-I38M succumbed to infection by 8 dpi (0% survival; 7-day mean TTD) (Figure 1(B and D)).

CA/147 replicated well in the lungs of mice at 3 and 5 dpi with titres reaching 7.2 and 8.1 log_10_TCID_50_/mL, respectively, except for one mouse with no evidence of infection (Figure 1(E and G)). Infectious virus was also found in all extrapulmonary organs: brain (4.2–7.3 log_10_TCID_50_/g), spleen (4.5–4.8 log_10_TCID_50_/g), and kidney (3.7–6.1 log_10_TCID_50_/g), at both time points.

Although CA/150 replicated well in mice lungs, titres were lower by 0.8–0.9 log_10_TCID_50_/g compared to CA/147; excluding one mouse with no evidence of infection (Figure 1(F and H); Table 4). Other signs of attenuation were the fewer number of animals with evidence of extrapulmonary infection and with lower titres in these organs.

**Table 4.** Baloxavir marboxil treatment of mice infected with clade 2.3.4.4b HPAI A(H5N1) viruses.

Virus^a^	Baloxavir dose (mg/kg)	Survival (%)	Wt. Loss (%)^b^	Mean TTD (day)	Virus titres (log_10_ TCID_50_/g)							
					3 dpi (n/total)				5 dpi (n/total)			
					Lung	Brain	Spleen	Kidney	Lung	Brain	Spleen	Kidney
CA/147 (Wildtype)	0	1/8 (12.5)	20	6	7.2 ± 0.2 (3/4)	4.2 ± 0.7 (3/4)	4.8 ± 0.4 (3/4)	3.7 ± 1.1 (3/4)	8.1 ± 0.3 (4 of 4)	7.3 ± 0.5 (4/4)	4.5 ± 0.6 (4/4)	6.1 ± 1.2 (4/4)
	5	7/8 (87.5)	5	11^c^	.	.	.	.	.	.	.	.
	15	8/8 (100)	6	.	.	.	.	.	.	.	.	.
	45	8/8 (100)	4	.	.	.	.	.	.	.	.	.
CA/150 (PA-I38M)	0	0/8 (0)	24	7	6.3 ± 0.6 (4/4)	2.1 (1/4)	2.9 ± 0.5 (3/4)	.	7.3 ± 0.3 (3/4)	5.9 ± 0.2 (3/4)	2.3 (1/4)	5.9 ± 0.2 (3/4)
	5	3/8 (37.5)	26	8	5.8 ± 0.8 (4/4)	2.1 (1/4)	.	.	5.0 ± 1.7 (3/4)	4.8; 4.8 (2/4)	.	.
	15	3/8 (37.5)	27	13	6.2; 4.2 (2/4)	.	.	.	5.2 ± 0.7 (4/4)	4.7 (1/4)	.	2.8 (1/4)
	45	5/8 (62.5)	14	11	.	.	.	.	2.3, 4.5 (2/4)	.	.	.

Six-week-old mice were inoculated with 5× MLD_50_ of A/California/147/2024 (CA/147, wildtype) or A/California/150/2024 (CA/150, PA-I38M). At 2 hpi, all inoculated mice were treated by oral gavage at the indicated baloxavir marboxil dose for 5 days given twice daily. Survival, weight loss, and time-to-death (TTD) were based on 8 mice per group. Separate set of animals (4 per time point) was euthanized at day 3 and 5 post-infection to determine infectious virus titres in tissues. Mice with undetectable virus titres were omitted from TCID_50_ calculations. HPAI, highly pathogenic avian influenza; TTD, within 14 (CA/147) or 21 (CA/150) days for mice that succumbed to infection or euthanized due to end-point.

^a^ Inoculum ∼30 TCID_50_ in 50 µL PBS (5× MLD_50_). CA/150 has three other differences in its genome (PB2-T58A, PB1-T6I, and NA-S369N) compared to CA/147.

^b^ Mean maximum weight loss.

^c^ Only one mouse succumbed to infection.

In mice infected with CA/147, early baloxavir treatment produced a potent antiviral effect. The survival rate for the 5 mg/kg-treated group was 87.5% (Table 4; Figure 1(A)). Baloxavir given at 15 and 45 mg/kg completely protected mice from lethality. Excluding the one mouse that succumbed to infection in the 5 mg/kg-treated group, none of the other animals lost more than 6% of initial weight (Figure 1(A and C)). Strikingly, infectious virus was not detected in any organs of the treated mice on either 3 or 5 dpi (Figure 1(E and G); Table 4).

In mice infected with CA/150 PA-I38M, baloxavir treatment at all doses did improve mean TTD. However, it did not protect all animals against lethality, even at the highest dose tested (62.5% survival) (Table 4; Figure 1(B)). On day 3, infectious virus was not detected in the 45 mg/kg-treated group, whereas two mice in the 15 mg/kg-treated group had virus in the lungs only. Moreover, virus replication was evident in all mice infected with CA/150 PA-I38M and treated with 5 mg/kg, which was not observed with the wildtype CA/147 virus.

One day after completion of baloxavir treatment (5 dpi), replication of the CA/150 PA-I38M virus in lungs was observed in all treatment groups, even at the highest dose tested. At lower doses, replication of CA/150 PA-I38M virus was also seen in other organs. Taken together, data obtained in the mouse model provide clear evidence for reduced susceptibility of clade 2.3.4.4b virus with PA-I38M.

## Discussion

Most clade 2.3.4.4b viruses collected from humans in the U.S. since October 2024 remained susceptible to U.S. FDA-approved influenza antivirals. One genotype D1.1 virus had the M2 blocker-resistance mutation M2-S31N, while another from genotype B3.13 had PA-I38M, which is associated with decreased susceptibility to baloxavir [30]. Both human cases had mild disease with conjunctivitis and recovered [6].

M2-S31N has been prevalent in certain influenza A viruses, including groups of HPAI A(H5N1) viruses [31,32]. Since our last report, its frequency among clade 2.3.4.4b animal viruses from this outbreak has increased from 0 to ∼1.3% [18]. Meanwhile, PA-I38M and PA-I38 T have been rare (<0.03%) natural polymorphisms among influenza viruses regardless of their origin [33,34]. Interestingly, the detection rate of these PA mutations was higher (0.2–0.4%) in clade 2.3.4.4.b viruses from animals in the U.S. [18, current study]. In the current report, some of the mutants were collected at the same locality among farm animals, including a small cluster of viruses containing PA-I38T collected from chickens, suggesting transmission among poultry.

The oseltamivir-resistance conferring NA-H275Y mutation was not found in viruses collected from human A(H5N1) cases and was detected in only three wild birds in the U.S. However, these NA-H275Y-containing viruses were not available for phenotypic testing. Notably, a cluster of A(H5N1) viruses with NA-H275Y was detected in Canada among poultry at several chicken farms in late 2024 [20], highlighting spread among poultry and the potential for transmission to exposed individuals. Influenza virus replicative fitness and transmissibility depend on NA activity. Therefore, we interrogated NA sequences for so-called “permissive mutations” that were previously shown to compensate for the detrimental effect of NA-H275Y on N1 enzyme activity and virus fitness. NA-H275Y viruses detected in the U.S. and Canada belong to genotype D1.1, all of which share NA-T289M, a substitution shown to partially compensate for the decreased NA expression and activity caused by NA-H275Y [35]. They also have NA-V234I and NA-N369S, whose effects are unknown; however, other substitutions at these residues were also associated with “permissive” effects [35–37]. Genotype B3.13 viruses had two known “permissive mutations,” NA-T289M and NA-R257K [35]. This sequence-based assessment was supported by our experiments with recNA proteins. Using small synthetic (MUNANA) and large natural (fetuin) substrates, we demonstrated that NA enzyme activity was not affected by the introduction of NA-H275Y in either B3.13 or D1.1 backgrounds.

We also used recNAs to determine the effect of mutations on susceptibility to NA inhibitors. This approach has been especially useful when viruses of interest are not available for testing [23,38]. As expected, NA-H275Y conferred HRI by oseltamivir and peramivir and had no effect on inhibition by zanamivir and laninamivir. Between NA-V116I and NA-S247N, two NA substitutions of interest found in human A(H5N1) cases and animal viruses, NA-S247N only slightly elevated oseltamivir IC_50_ in both genetic backgrounds, similar to what was seen in genotype B1.2 [18]. Modestly elevated (3- to 22-fold) oseltamivir IC_50_s were also observed for other mutations. Not surprisingly, the five mutations that conferred RI/HRI by zanamivir (e.g., T438I) also resulted in lower NA activity, which is in accordance with the minimalist approach used to design this drug: the closer the inhibitor is to the natural substrate the less likely the targeted protein can mutate without loss of its natural function [39].

The new mutation NA-I427T conferred RI (90-fold) by zanamivir and only slightly elevated oseltamivir IC_50_ (6-fold), which, in accordance with the cut-off set for surveillance purposes, is interpreted as “normal inhibition.” This testing outcome could be misconstrued to mean that oseltamivir would be a more potent inhibitor against this mutant; however, there is no apparent difference in IC_50_s for these two inhibitors (22 vs 28 nM). This discordance stems from the higher oseltamivir IC_50_ baseline for wildtype clade 2.3.4.4b viruses [18,19]. While interpretation of NI data should be conducted with caution, testing in cell culture has proven to be unreliable and is not used to assess susceptibility to this class of antivirals. On the other hand, cell culture-based assays are commonly used to assess susceptibility to other antivirals, including baloxavir.

Using the single-cycle replication assay IRINA [18,27], we showed that baloxavir susceptibilities of B3.13 and D1.1 viruses were similar to those of seasonal influenza A viruses. In humans, a single-dose baloxavir marboxil is used for early treatment of uncomplicated influenza in outpatients. A phase 3 trial reported that emergence of PA-I38 T/M substitutions was associated with longer duration of symptoms than in baloxavir-treated patients whose viruses did not contain these substitutions [40]. However, laboratory correlates of clinically-relevant resistance to baloxavir have not yet been established. For the purpose of virological surveillance, reduced baloxavir susceptibility is provisionally defined as greater than 3-fold rise over EC_50_ of PA-sequence matched wildtype virus or a subtype-specific median. PA-I38T, the principal marker of baloxavir resistance, has been associated with an 11- to 614-fold rise, depending on the genetic background, assay, and other variables [41]. In IRINA, PA-I38T conferred ∼70- to 100-fold rise in baloxavir EC_50_, whereas PA-I38M led to 9- to 17-fold [18, current study]. Unlike for PA-I38T [42–44], the impact of PA-I38M on the effectiveness of baloxavir treatment in animal models has not been investigated prior to this study. Using a mouse model, we showed that a 5-day treatment course with baloxavir (marboxil) efficiently suppressed replication of the wildtype CA/147 virus in lungs and other organs collected on 3 and 5 dpi, even at 5 mg/kg, the lowest dose tested. Conversely, the CA/150 PA-I38M virus clearly displayed reduced baloxavir susceptibility, as evidenced by survival, TTD, body weight, and virus titres. This indicates that the 17-fold rise in EC_50_ was associated with reduced therapeutic effect of baloxavir in this model. Mutations that result in higher fold-rises may have more drastic effects, as was reported for A(H1N1) viruses with PA-I38T substitution that were tested in mice [42,44]. Further investigations are needed to establish a correlation between EC_50_ values and baloxavir treatment outcomes *in vivo*. It is worth noting that after completion of treatment in our study, some mice infected with the CA/150 PA-I38M virus became severely lethargic, developed neurological signs, and eventually died or were euthanized, indicating virus rebound. It is tempting to speculate that the survival rate of PA-I38M virus-infected animals could be improved if baloxavir treatment was prolonged beyond five days. Despite the well-recognized difference in pharmacokinetics of baloxavir between humans and laboratory animals [28,29, 45–47], it is reasonable to expect viruses with reduced susceptibility in laboratory animal models to be less responsive to baloxavir treatment in humans.

For risk assessment purposes, it is beneficial to know whether mutations that reduce antiviral susceptibility also affect the replicative fitness of influenza viruses. In this study, the clade 2.3.4.4b virus with PA-I38M replicated well in MDCK and two other commonly used laboratory cell lines, while being only slightly attenuated in mice, as seen by the extended TTD and up to 2-log lower virus titres. *In vitro*, a modest attenuation of replication by PA-I38M was reported in a recombinant A/Hong Kong/483/1997 (H5N1) virus background [48]. However, most seasonal influenza viruses with PA-I38M or PA-I38T showed little or no attenuation in cell culture, mice, or ferrets [42,43,49].

One of our study’s limitations was the use of a wildtype virus that had three additional amino acid differences in the genome. While our findings support PA-I38M as a marker for reduced susceptibility of the clade 2.3.4.4b HPAI A(H5N1) virus in a mouse model, the possible influence of the other genomic differences on viral fitness cannot be ruled out. Another limitation of this study was the incomplete viral genome sequences for some human cases, particularly for those with low viral load specimens [6], as well as unsuccessful attempts to isolate some viruses for phenotypic testing. For viruses with incomplete genome sequences, their genotypes were inferred based on sequence similarities to other viruses identified in animals that the case patient may have been exposed to, or collected in the same setting (i.e. culling or depopulation operation) as other cases whose complete virus genomes were analyzed [6]. Among the viruses that were not isolated were those that had NA mutation of interest (e.g. V116I, S247N). To increase the likelihood of virus recovery, various cell culture systems (e.g. MDCK-SIAT1) should be considered for virus isolation. The use of alternative systems to assess antiviral susceptibility could also be instrumental, such as in the use of recNA proteins.

Animal models offer an opportunity to evaluate the emergence of resistance following antiviral treatment. However, in our study, the drastic reduction in the wildtype B3.13 virus replication to undetectable levels in mice treated with baloxavir precluded analysis of emerging virus subpopulations. A similar reduction was reported for B3.13-infected mice orally treated with 50 mg/kg baloxavir (marboxil) for 5 days [50]. In both studies, this potent antiviral effect on virus replication was observed when baloxavir treatment was initiated early. However, when treatment was delayed by 24–72 h, there was emergence of the PA-I38T substitution [41]. In another mouse study with B3.13 virus and treatment delayed by 24 h, PA-I38T was found to emerge following a single dose of baloxavir (acid) given subcutaneously [51].

Overall, the results of our study highlight the importance of continuing to monitor antiviral drug susceptibilities of clade 2.3.4.4b HPAI A(H5N1) viruses from animals and infected persons using sequence analyses, *in vitro* assays, and animal models to inform public health recommendations on the use of antivirals. Viruses with decreased susceptibility to approved antivirals emerge spontaneously in nature and can spill over to humans. Therefore, early initiation of antiviral treatment, prolonged regimens, and higher doses of antivirals may be necessary to control such zoonotic infections. A combination of antivirals with different mechanisms of action could be considered, particularly for treatment of hospitalized or immunocompromised patients [11]. Indeed, combination antiviral treatment was administered to two of the hospitalized patients in the US [6] and a critically ill adolescent in Canada [14]. When confronting infections caused by HPAI A(H5N1) viruses, combination therapy should be considered because these viruses may contain spontaneously acquired mutations that make them less susceptible to monotherapy as was observed in this study where the CA/150 virus, which contains PA-I38M, showed reduced susceptibility to baloxavir treatment *in vivo*.

## Supplementary Material

Pascua_Update_H5_SuppMat_TEMi_2025_1441_R2-clean.docx

Pascua_Update_H5_SuppFig_TEMi_2025_1441_R2.pdf
