# Supplementary material for: Antiviral susceptibility of clade 2.3.4.4b highly pathogenic avian influenza A(H5N1) viruses from humans in the United States, October 2024 to February 2025
Source: Emerg Microbes Infect. 2025 Dec 15;15(1):2601372. doi: 10.1080/22221751.2025.2601372 (PMC12777753; doi:10.1080/22221751.2025.2601372)
Supplement: Pascua_Update_H5_SuppMat_TEMi_2025_1441_R2-clean.docx [file TEMI_A_2601372_SM9201.docx]

**Antiviral susceptibility of clade 2.3.4.4b highly pathogenic avian influenza A(H5N1) viruses from humans in the United States, October 2024 to February 2025**

**Supplementary Information**

**Supplementary Figure legends**

**Figure S1.** *In vitro* replicative fitness of viruses with or without PA-I38M substitution. MDCK, MDCK-SIAT1 cells, and humanized MDCK (hCK) cell monolayers were infected at a multiplicity of infection (MOI) of 0.00005. Supernatants were collected at 8, 12, 24, 48, and 72 h post-infection and infectious virus titers were determined and expressed as log_10_TCID_50_/mL. The lower limit of virus detection is 1.50 log_10_ TCID_50_/mL. Data shown are mean ± SD from two experiments in duplicates. Two-way ANOVA with Sidak’s multiple comparison *post-hoc* test was used for statistical comparisons (**p*˂0.05; ***p*=0.01; ****p*=001).

**Figure S2.** Determination of 50% lethal doses of A/California/147/2024 A(H5N1) and A/California/150/2024 A(H5N1) viruses in mice (MLD_50_). BALB/c mice (female, 6-week-old, n = 8 animals per virus inoculum dose) were deeply anaesthetized and intranasally inoculated with ten-fold serial dilutions of A/California/147 (CA/147, wildtype) or A/California/150/2024 (CA/150, PA-I38M mutant) virus in 50 μL of PBS. Survival (A, B) and body weights (C, D) were monitored daily for 14 days (n=8 per inoculum dose). Data are mean percentages ± SD of the starting weight.

**Table S1.** Confirmed human cases of clade 2.3.4.4b HPAI A(H5N1) infection in the United States, October 2024 - February 2025.

| **Influenza A(H5N1) virus** | **Date collected** | **Exposure** | **Genome sequencing** | **Genotype** | **GISAID isolate ID** | **Originating lab/submitter institution** |
| --- | --- | --- | --- | --- | --- | --- |
| **A/California/135/2024** | 10/1/2024 | Cow | Complete | B3.13 | EPI_ISL_19463618 | California Department of Health Services/CDC-Atlanta |
| **A/California/146/2024** | 10/2/2024 | Cow | Complete | B3.13 | EPI_ISL_19473581 | California Department of Health Services/CDC-Atlanta |
| **A/California/147/2024** | 10/7/2024 | Cow | Complete | B3.13 | EPI_ISL_19481947 | California Department of Health Services/CDC-Atlanta |
| **A/California/148/2024** | 10/8/2024 | Cow | Complete | B3.13 | EPI_ISL_19481948 | California Department of Health Services/CDC-Atlanta |
| A/California/149/2024 | 10/7/2024 | Cow | Partial | B3.13 | EPI_ISL_19481949 | California Department of Health Services/CDC-Atlanta |
| **A/California/150/2024** | 10/9/2024 | Cow | Complete | B3.13 | EPI_ISL_19544645 | California Department of Health Services/CDC-Atlanta |
| **A/California/151/2024** | 10/9/2024 | Cow | Complete | B3.13 | EPI_ISL_19531293 | California Department of Health Services/CDC-Atlanta |
| **A/California/152/2024** | 10/10/2024 | Cow | Complete | B3.13 | EPI_ISL_19497981 | California Department of Health Services/CDC-Atlanta |
| **A/California/153/2024** | 10/9/2024 | Cow | Complete | B3.13 | EPI_ISL_19497980 | California Department of Health Services/CDC-Atlanta |
| A/California/154/2024 | 10/9/2024 | Cow | No | B3.13 | VNR | California Department of Health Services/CDC-Atlanta |
| **A/California/155/2024** | 10/16/2024 | Cow | Complete | B3.13 | EPI_ISL_19512043 | California Department of Health Services/CDC-Atlanta |
| A/California/156/2024 | 10/16/2024 | Cow | No | B3.13 | VNR | California Department of Health Services/CDC-Atlanta |
| A/California/167/2024 | 10/17/2024 | Cow | No | B3.13 | VNR | California Department of Health Services/CDC-Atlanta |
| A/California/168/2024 | 10/18/2024 | Cow | Complete | B3.13 | EPI_ISL_19512044 | California Department of Health Services/CDC-Atlanta |
| A/California/169/2024 | 10/22/2024 | Cow | No | B3.13 | VNR | California Department of Health Services/CDC-Atlanta |
| A/California/171/2024 | 10/25/2024 | Cow | Partial | B3.13 | EPI_ISL_19531294 | California Department of Health Services/CDC-Atlanta |
| A/California/172/2024 | 10/24/2024 | Cow | Partial | B3.13 | EPI_ISL_19531295 | California Department of Health Services/CDC-Atlanta |
| **A/California/173/2024** | 10/24/2024 | Cow | Complete | B3.13 | EPI_ISL_19531296 | California Department of Health Services/CDC-Atlanta |
| A/California/174/2024 | 10/30/2024 | Cow | Partial | B3.13 | EPI_ISL_19531297 | California Department of Health Services/CDC-Atlanta |
| A/California/179/2024 | 10/31/2024 | Cow | No | B3.13 | VNR | California Department of Health Services/CDC-Atlanta |
| A/California/180/2024 | 11/7/2024 | Cow | Partial | B3.13 | EPI_ISL_19590701 | California Department of Health Services/CDC-Atlanta |
| A/California/181/2024 | 11/7/2024 | Cow | Complete | B3.13 | EPI_ISL_19590702 | California Department of Health Services/CDC-Atlanta |
| A/California/182/2024 | 11/7/2024 | Cow | Partial | B3.13 | EPI_ISL_19590703 | California Department of Health Services/CDC-Atlanta |
| A/California/183/2024 | 11/7/2024 | Cow | Complete | B3.13 | EPI_ISL_19590704 | California Department of Health Services/CDC-Atlanta |
| A/California/190/2024 | 11/12/2024 | Cow | Partial | B3.13 | EPI_ISL_19590706 | California Department of Health Services/CDC-Atlanta |
| A/California/191/2024 | 11/13/2024 | Cow | Complete | B3.13 | EPI_ISL_19590707 | California Department of Health Services/CDC-Atlanta |
| A/California/192/2024 | 11/12/2024 | Unknown | Partial | B3.13 | EPI_ISL_19597300 | California Department of Health Services/CDC-Atlanta |
| A/California/193/2024 | 11/18/2024 | Cow | Complete | B3.13 | EPI_ISL_19590708 | California Department of Health Services/CDC-Atlanta |
| A/California/194/2024 | 11/22/2024 | Cow | Complete | B3.13 | EPI_ISL_19616453 | California Department of Health Services/CDC-Atlanta |
| A/California/195/2024 | 11/23/2024 | Cow | Complete | B3.13 | EPI_ISL_19616451 | California Department of Health Services/CDC-Atlanta |
| A/California/196/2024 | 11/27/2024 | Cow | Partial | B3.13 | EPI_ISL_19616452 | California Department of Health Services/CDC-Atlanta |
| A/California/213/2024 | 12/6/2024 | Cow | Complete | B3.13 | EPI_ISL_19628008 | California Department of Health Services/CDC-Atlanta |
| A/California/214/2024 | 12/6/2024 | Cow | Partial | B3.13 | EPI_ISL_19628009 | California Department of Health Services/CDC-Atlanta |
| A/California/215/2024 | 12/10/2024 | Cow | Complete | B3.13 | Pending | California Department of Health Services/CDC-Atlanta |
| A/California/216/2024 | 12/10/2024 | Cow | Complete | B3.13 | EPI_ISL_19660248 | California Department of Health Services/CDC-Atlanta |
| A/California/217/2024 | 12/19/2024 | Cow | No | B3.13 | VNR | California Department of Health Services/CDC-Atlanta |
| A/California/227/2024 | 12/16/2024 | Unknown | Complete | B3.13 | EPI_ISL_20143473 | California Department of Health Services/CDC-Atlanta |
| **A/Iowa/124/2024** | 12/17/2024 | Poultry | Complete | D1.1 | EPI_ISL_19669941 | Iowa State Hygienic Laboratory/CDC-Atlanta |
| A/Louisiana/12/2024 | 12/11/2024 | Poultry | Complete | D1.1 | EPI_ISL_19634828 | Louisiana Department of Health and Hospitals/CDC-Atlanta |
| **A/Nevada/10/2025** | 2/4/2025 | Cow | Complete | D1.1 | EPI_ISL_19726293 | Nevada State Health Laboratory/CDC-Atlanta |
| A/Oregon/255/2024 | 11/5/2024 | Poultry | Partial | D1.1 | EPI_ISL_19590705 | Oregon Public Health Laboratory/CDC-Atlanta |
| A/Washington/236/2024 | 10/18/2024 | Poultry | No | D1.1 | VNR | Washington State Public Health Lab/CDC-Atlanta |
| **A/Washington/239/2024** | 10/18/2024 | Poultry | Partial | D1.1 | EPI_ISL_19531298 | Washington State Public Health Lab/CDC-Atlanta |
| **A/Washington/240/2024** | 10/18/2024 | Poultry | Complete | D1.1 | EPI_ISL_19531299 | Washington State Public Health Lab/CDC-Atlanta |
| A/Washington/243/2024 | 10/18/2024 | Poultry | No | D1.1 | VNR | Washington State Public Health Lab/CDC-Atlanta |
| A/Washington/247/2024 | 10/18/2024 | Poultry | No | D1.1 | EPI_ISL_19512047 | Washington State Public Health Lab/CDC-Atlanta |
| A/Washington/249/2024 | 10/19/2024 | Poultry | No | D1.1 | VNR | Washington State Public Health Lab/CDC-Atlanta |
| A/Washington/251/2024 | 10/24/2024 | Poultry | Partial | D1.1 | EPI_ISL_19531300 | Washington State Public Health Lab/CDC-Atlanta |
| A/Washington/252/2024 | 10/24/2024 | Poultry | Partial | D1.1 | EPI_ISL_19531301 | Washington State Public Health Lab/CDC-Atlanta |
| A/Washington/253/2024 | 10/24/2024 | Poultry | Partial | D1.1 | EPI_ISL_19531302 | Washington State Public Health Lab/CDC-Atlanta |
| **A/Washington/254/2024** | 10/23/2024 | Poultry | Complete | D1.1 | EPI_ISL_19666173 | Washington State Public Health Lab/CDC-Atlanta |
| **A/Washington/255/2024** | 10/29/2024 | Poultry | Complete | D1.1 | EPI_ISL_19552697 | Washington State Public Health Lab/CDC-Atlanta |
| A/Wisconsin/179/2024 | 12/13/2024 | Poultry | Partial | D1.1 | EPI_ISL_19639064 | Wisconsin State Laboratory of Hygiene/CDC-Atlanta |
| **A/Wyoming/01/2025** | 2/11/2025 | Cow | Complete | D1.1 | EPI_ISL_19749443 | Colorado Department of Health Lab/CDC-Atlanta |
| A/Ohio/06-1/2025 | 2/12/2025 | Poultry | Complete | D1.3 | EPI_ISL_19785793 | Ohio Department of Health Lab/CDC-Atlanta |

Viruses were recovered from specimens collected prior to antiviral treatment. Viruses available for testing are indicated in **bold** text. A complete whole genome sequence is required to assign genotype. The genotype of viruses with incomplete genome sequence was inferred based on sequence similarities to other viruses identified in animals to which the case patient was exposed to or identified in the same setting (i.e., culling or depopulation operation) where other cases had complete virus genomes analyzed [1]. HPAI, highly pathogenic avian influenza; VNR, virus not recovered.

**Table S2.** Genotypes defined by GenoFLU (<https://github.com/USDA-VS/GenoFLU>).

| Introduction | Genotype | Reported name | Segments | | | | | | | |
| --- | --- | --- | --- | --- | --- | --- | --- | --- | --- | --- |
|  |  |  | PB2 | PB1 | PA | HA | NP | NA | M | NS |
| A3 | A3 | EA 2.3.4.4b A(H5N1) (A3) | ea3 | ea3 | ea3 | ea3 | ea3 | ea3 | ea3 | ea3 |
| A1 | B1.1 | EA/AM 2.3.4.4b A(H5N1) (B1.1) | am1.1 | am1.1 | ea1 | ea1 | am1.2 | ea1 | ea1 | ea1 |
| A1 | **B3.13** | EA/AM 2.3.4.4b A(H5N1) (B1.13) | am2.2 | am4 | ea1 | ea1 | am8 | ea1 | ea1 | am1.1 |
| A3 | **D1.1** | EA/AM 2.3.4.4b AH5N1) (D1.1) | am24 | ea3 | am4 | ea3 | am13 | am4 | ea3 | ea3 |
| A3 | **D1.3** | EA/AM 2.3.4.4b A(H5N1) (D1.3) | am24 | ea3 | am4 | ea3 | am13 | ea3 | ea3 | ea3 |

Genotypes of highly pathogenic avian influenza A(H5N1) viruses recovered from human cases in the U.S. are in **bold** text. These genotypes differ in the gene segments encoding antiviral-targeted viral proteins (M2, NA, and PA). Underlined text indicates introduction of new lineage for each segment through reassortment.

**Table S3.** Sequence differences in M2, NA, and PA proteins of genotype B3.13 HPAI A(H5N1) viruses collected in the U.S., October 2024 - February 2025.

| Influenza A(H5N1) virus^a^ | M2 | | | | | NA (head domain)^b^ | | | | | | | | | | | | | PA | | | | | | | | | | | | | | | |
| --- | --- | --- | --- | --- | --- | --- | --- | --- | --- | --- | --- | --- | --- | --- | --- | --- | --- | --- | --- | --- | --- | --- | --- | --- | --- | --- | --- | --- | --- | --- | --- | --- | --- | --- |
|  | 13 | 52 | 61 | 82 | 88 | 84 | 94 | 111 | **116** | 122 | 143 | 260 | 269 | 272 | 321 | 339 | 369 | **439** | **38^c^** | 68 | 99 | 113 | 219 | 274 | 277 | 305 | 308 | 432 | 486 | 497 | 557 | 558 | 655 |  |
| A/bald eagle/FL/2022 (B1.1) | N | Y | G | S | D | T | I | K | V | I | K | K | L | P | V | S | S | S | I | P | G | K | L | P | S | Y | I | V | I | K | V | S | L |  |
| **A/California/135/2024** | . | . | . | . | N | . | . | . | . | . | . | . | M | . | I | P | . | G | . | . | . | R | I | . | P | . | . | I | . | R | . | L | . |  |
| **A/California/146/2024** | . | . | . | . | N | . | . | . | . | . | . | . | M | . | I | P | . | G | . | . | . | R | I | . | P | . | . | I | . | R | . | L | . |  |
| **A/California/147/2024** | . | . | . | . | N | . | . | . | . | . | . | . | M | . | I | P | . | . | . | . | . | R | I | . | P | . | . | I | . | R | . | L | . |  |
| **A/California/148/2024** | . | . | . | . | N | . | . | . | . | . | . | . | M | . | I | P | . | . | . | . | . | R | I | . | P | . | . | I | . | R | . | L | . |  |
| A/California/149/2024 |  |  |  |  |  | . | . | . | . | . | . | . | M | . | I | P | . | . |  |  |  |  |  |  |  |  |  |  |  |  |  |  |  |  |
| **A/California/150/2024** | . | . | . | . | N | . | . | . | . | . | . | . | M | . | I | P | N | . | **M** | . | . | R | I | . | P | . | . | I | . | R | . | L | . |  |
| **A/California/151/2024** | . | . | . | . | N | . | . | . | . | . | . | . | M | . | I | P | . | . | . | . | . | R | I | . | P | . | . | I | . | R | . | L | . |  |
| **A/California/152/2024** | . | . | . | . | N | . | . | . | . | . | . | . | M | . | I | P | . | . | . | S | . | R | I | . | P | . | . | I | . | R | . | L | . |  |
| **A/California/153/2024** | . | . | . | . | N | . | . | . | . | . | . | . | M | . | I | P | . | . | . | S | . | R | I | . | P | . | . | I | . | R | . | L | . |  |
| A/California/154/2024 |  |  |  |  |  |  |  |  |  |  |  |  |  |  |  |  |  |  |  |  |  |  |  |  |  |  |  |  |  |  |  |  |  |  |
| **A/California/155/2024** | . | . | . | . | N | . | . | . | . | V | V | . | M | . | I | P | . | . | . | S | . | R | I | . | P | . | . | I | . | R | . | L | F |  |
| A/California/156/2024 |  |  |  |  |  |  |  |  |  |  |  |  |  |  |  |  |  |  |  |  |  |  |  |  |  |  |  |  |  |  |  |  |  |  |
| A/California/167/2024 |  |  |  |  |  |  |  |  |  |  |  |  |  |  |  |  |  |  |  |  |  |  |  |  |  |  |  |  |  |  |  |  |  |  |
| A/California/168/2024 | . | . | . | . | N | A | . | . | . | . | . | . | M | . | I | P | . | . | . | . | . | R | I | . | P | . | . | I | . | R | . | L | . |  |
| A/California/169/2024 | . | . | . | . | N |  |  |  |  |  |  | . | M | . | I | P | . | . |  |  |  |  |  |  |  |  |  |  |  |  |  |  |  |  |
| A/California/171/2024 |  |  |  |  |  | . | . | . | . | . | . | R | M | . | I | P | . | . |  |  |  |  |  |  |  |  |  |  |  |  |  |  |  |  |
| A/California/172/2024 |  |  |  |  |  | . | . | . | . | . | . | . | V | . | I | P | . | . |  |  |  |  |  |  |  |  |  |  |  |  |  |  |  |  |
| **A/California/173/2024** | . | . | . | . | N | . | . | . | . | . | . | . | V | . | I | P | . | . | . | . | . | R | I | . | P | . | . | I | . | R | . | L | . |  |
| A/California/174/2024 | . | . | . | . | N | . | . | . | . | . | . | . | M | . | I | P | . | . |  |  |  |  |  |  |  |  |  |  |  |  |  |  |  |  |
| A/California/179/2024 |  |  |  |  |  | . | V | . | . | . | . | . | M | . | I | P | . | . |  |  |  |  |  |  |  |  |  |  |  |  |  |  |  |  |
| A/California/180/2024 |  |  |  |  |  |  |  |  |  |  |  | . | M | . | I | P | . | . |  |  |  |  |  |  |  |  |  |  |  |  |  |  |  |  |
| A/California/181/2024 | . | . | . | . | N | . | . | . | . | . | . | . | M | . | I | P | . | . | . | S | . | R | I | . | P | . | . | I | M | R | . | L | F |  |
| A/California/182/2024 |  |  |  |  |  | . | . | . | . | . | . | . | M | . | I | P | . | . |  |  |  |  |  |  |  |  |  |  |  |  |  |  |  |  |
| A/California/183/2024 | . | . | . | . | N |  |  |  |  |  |  | . | M | . | I | P | . | . | . | S | . | R | I | . | P | . | . | I | . | R | . | L | F |  |
| A/California/190/2024 | . | . | . | . | N | . | . | . | . | . | . | . | M | . | I | P | . | . |  |  |  |  |  |  |  |  |  |  |  |  |  |  |  |  |
| A/California/191/2024 | . | . | . | . | N | . | . | . | . | . | . | . | M | . | I | P | . | . | . | . | I | R | I | . | P | . | . | I | . | R | . | L | . |  |
| A/California/192/2024 |  |  |  |  |  | . | . | E | . | . | . | . | M | . | I | P | . | . |  |  |  |  |  |  |  |  |  |  |  |  |  |  |  |  |
| A/California/193/2024 | S | . | . | . | N | . | . | . | . | . | . | . | M | . | I | P | . | . | . | . | . | R | I | . | P | . | V | I | . | R | . | L | . |  |
| A/California/194/2024 | . | . | . | . | N | . | . | . | . | . | . | . | M | . | I | P | . | . | . | . | . | R | I | . | P | . | . | I | . | R | . | L | . |  |
| A/California/195/2024 | S | . | . | . | N | . | . | . | . | . | . | . | M | . | I | P | . | . | . | . | . | R | I | . | P | . | V | I | . | R | I | L | . |  |
| A/California/196/2024 | S | . | . | . | N | . | . | . | . | . | . | . | M | . | I | P | . | . |  |  |  |  |  |  |  |  |  |  |  |  |  |  |  |  |
| A/California/213/2024 | . | . | . | . | N | . | . | . | . | . | . | . | M | . | I | P | . | . | . | S | . | R | I |  | P | H | . | I | . | R | . | L | F |  |
| A/California/214/2024 | . | . | . | . | N | . | . | . | . | . | . | . | M | . | I | P | . | . |  |  |  |  |  |  |  |  |  |  |  |  |  |  |  |  |
| A/California/215/2024 | . | . | . | N | N | . | . | . | I | . | . | . | M | . | I | P | . | . | . | S | . | R | I | . | P | . | . | I | . | R | . | L | F |  |
| A/California/216/2024 | . | . | . | . | N | . | . | . | . | . | . | . | M | . | I | P | . | . | . | S | . | R | I | L | P | . | . | I | . | R | . | L | . |  |
| A/California/217/2024 |  |  |  |  |  |  |  |  |  |  |  |  |  |  |  |  |  |  |  |  |  |  |  |  |  |  |  |  |  |  |  |  |  |  |
| A/California/227/2025 | . | C | . | . | N | . | . | . | . | . | . | . | M | S | I | P | . | . | . | S | . | R | I | . | P | . | . | I | . | R | . | L | F |  |

Residues shaded in black indicate unavailable sequence information. HPAI, highly pathogenic avian influenza; NA, neuraminidase; PA, polymerase acidic.

^a^Viruses in **bold** were available for testing.

^b^Residues with substitution of interest in the NA head domain are indicated in **bold**.

^c^PA substitution in red text (I38M) confers reduced baloxavir susceptibility.

**Table S4.** Sequence differences in M2, NA, and PA proteins of genotype D1.1 and D1.3 HPAI A(H5N1) viruses collected in the U.S., October 2024 - February 2025.

Residues shaded in black indicate unavailable sequence information. HPAI, highly pathogenic avian influenza; NA, neuraminidase; PA, polymerase acidic.^a^Viruses in **bold** were available for testing.

^b^M2 substitution in red text (S31N) confers cross-resistance to amantadine and rimantadine.

^c^Residues with substitution of interest in the NA head domain are indicated in **bold**.

**Table S5**. Flagged M2, NA, and PA substitutions in clade 2.3.4.4b HPAI A(H5N1) viruses collected from animal hosts in the U.S., October 2024 – February 2025.

| **Amino acid substitution** | **Region/functional role** | **Animal host** | | **Count** | **Rationale for flagging^a^** |
| --- | --- | --- | --- | --- | --- |
| **M2 (n=5,536 sequences)** | | |  | 78 (1.4%) |  |
| L26F | TMD | poultry | | 1 | Resistance [2] |
| V27A | TMD | wild bird, poultry, cattle | | 10 | Resistance [2] |
| A30T | TMD | wild bird, poultry | | 4 | Resistance [2] |
| **S31N** | TMD | wild bird, poultry | | 63 | Resistance [2] |
| **NA (n=5,382 sequences)^b^** | | |  | 347 (6.5%) |  |
| S110F (110) | - | poultry | | 2 | In A(H1N1)pdm09 confers RI [3] |
| S110Y+V116I (110+116) | - | poultry | | 5 | New substitution at 110; S110F in A(H1N1)pdm09 confers RI [3] |
| **V116A (116)** | - | wild bird, poultry | | 19 | In A(H5N1) confers RI [3] |
| **V116I (116)** | - | wild bird, poultry, cow | | 124 | New substitution at 116; V116A in A(H5N1) confers RI [3] |
| V116F (116) | - | wild bird | | 5 | New substitution at 116; V116A in A(H5N1) confers RI [3] |
| V116A+G147R (116+147) | - | wild bird | | 1 | New combination of substitutions; V116A in A(H5N1) confers RI [3] |
| I117L (117) | - | poultry | | 1 | New substitution; I117R in A(H1N1)pdm09 confers RI [3] |
| I117M (117) | - | poultry, cow | | 7 | New substitution; I117R in A(H1N1)pdm09 confers RI [3] |
| I117N (117) | - | poultry | | 3 | New substitution; I117R in A(H1N1)pdm09 confers RI [3] |
| R118S (118) | catalytic | wild bird | | 10 | Catalytic residue in the NA active site with unknown effect |
| E119D (119) | framework | wild bird | | 1 | In A(H5N1) confers RI/HRI [3] |
| Q136H (136) | - | wild bird | | 17 | New substitution; Q136L in A(H5N1) confers RI/HRI [3] |
| G147E (147) | - | wild bird | | 4 | New substitution in 150-loop; in type B confers RI [3] |
| G147R (147) | - | wild bird | | 1 | New substitution in 150-loop; G147E in type B confers RI [3] |
| V149G (149) | - | wild bird | | 1 | New substitution in 150-loop of NA with unknown effect |
| V149I (149) | - | wild bird, poultry | | 8 | In B3.1 confers normal inhibition [4] |
| R152G (152) | catalytic | poultry | | 2 | New substitution; R152K in A(H1N1)pdm09 confers RI [3] |
| R152I (152) | catalytic | poultry | | 1 | New substitution; R152K in A(H1N1)pdm09 confers RI [3] |
| R156K (156) | framework | wild bird, poultry | | 3 | Substitution at framework residue with unknown effect [5] |
| D199G (198) | framework | wild bird | | 1 | In A(H5N1 confers RI [3] |
| I223M (222) | framework | poultry | | 1 | In A(H5N1) confers RI [3] |
| I223T (222) | framework | wild bird | | 1 | In A(H1N1)pdm09 confers RI [3] |
| I223V (222) | framework | wild bird, poultry | | 2 | Can enhance effect of H275Y [3] |
| E228D (227) | framework | wild bird | | 1 | Substitution at framework residue in the active site with unknown effect |
| S247G (246) | **-** | wild bird, poultry, cow | | 5 | In A(H1N1)pdm09 confers RI [3] |
| **S247N (246)** | - | wild bird, poultry, cow | | 6 | In A(H5N1) confers RI [3] |
| S247R (246) | **-** | wild bird | | 1 | In A(H1N1)pdm09 confers RI/HRI [3] |
| **H275Y (274)** | framework | wild bird | | **3** | In A(H5N1) confers RI/HRI^c^ [3] |
| H275N (274) | framework | wild bird | | 1 | New substitution; H275Y in A(H5N1) confers RI/HRI [3] |
| H275Q (274) | framework | wild bird | | 1 | New substitution; H275Y in A(H5N1) confers RI/HRI [3] |
| N295D (294) | framework | wild bird | | 5 | In A(H5N1) confers normal inhibition in B1.1 [3] |
| R368K (371) | catalytic | wild bird | | 1 | Substitution at catalytic residue; in A(H3N2) confers RI [3] |
| E425G (425) | framework | wild bird | | 1 | Substitution at framework residue with unknown effect |
| I427F (427) | - | wild bird | | 1 | New substitution; I427T In A(H1N1)pdm09 confers RI [3] |
| I427S (427) | - | wild bird | | 1 | New substitution; I427T In A(H1N1)pdm09 confers RI [3] |
| **I427T (427)** | **-** | wild bird, poultry | | 12 | In A(H1N1)pdm09 confers RI [3] |
| I427V (427) | - | poultry | | 1 | In swine-origin A(H1N1) variant virus confers RI [6] |
| K432E (432) | - | wild bird | | 1 | In A(H5N1) confers RI [7] |
| K432Q (432) | - | wild bird, poultry | | 2 | New substitution; K432E/T in A(H5N1) confers RI [3,7] |
| **K432R (432)** | - | wild bird, poultry, cat | | 43 | New substitution; K432E/T in A(H5N1) confers RI [3,7] |
| T438A (439) | - | cow | | 11 | New substitution (T438I in A(H5N1) confers RI/HRI [3] |
| **T438I (439)** | **-** | wild bird | | 8 | In A(H5N1), confers RI/HRI [3] |
| **S439G (440)** | - | wild bird, poultry, cow | | 22 | New substitution; substitution at adjacent T438 confers RI/HRI [3] |
| **PA (n=5,090 sequences)** | | |  | 55 (1.1%) |  |
| E18G | CEN | wild bird, poultry, cow | | 5 | In A(H1N1), confers reduced susceptibility [8] |
| E18K | CEN | wild bird, poultry | | 6 | New substitution; E18G in A(H1N1) confers reduced susceptibility [8] |
| A36S | CEN | poultry | | 3 | New substitution; A36V in A(H1N1) confers reduced susceptibility [8] |
| A36T | CEN | wild bird, poultry, cat | | 7 | New substitution; A36V in A(H3N2) confers reduced susceptibility [7] |
| A36V | CEN | skunk | | 1 | In A(H3N2) confers reduced susceptibility [8] |
| A37S | CEN | wild bird, poultry, cat | | 4 | New substitution; A37T in A(H5N1) confers reduced susceptibility [8] |
| **I38M** | CEN | wild bird, poultry, cow | | 5 | Confers reduced susceptibility [8] |
| I38T | CEN | wild bird, poultry, cat, cow | | 13 | Confers reduced susceptibility^d^ [8] |
| I38V | CEN | wild bird, poultry, cow | | 7 | In A(H1N1), minimal to effect on susceptibility [7] |
| V122A | CEN | poultry | | 3 | Confers reduced susceptibility [9] |

Sequences of animal viruses collected from October 1, 2024, to February 28, 2025 were analyzed (sequences submitted by June 23, 2025). Count includes mixtures (e.g., PA-I38M/I and PA-I38T/I). Flagged M2, NA, and PA substitutions in **bold** were tested using available human A(H5N1) viruses (Table 1 and 2) or recNA proteins (Table 3). CEN, cap-dependent endonuclease domain; HPAI, highly pathogenic avian influenza; NA, neuraminidase; PA, polymerase acidic; TMD, transmembrane domain.

^a^Residues and substitutions were flagged due to their presence in functional region of proteins and/or previous association with reduced inhibition/susceptibility to approved antiviral agents.

^b^Corresponding N2 numbering are in parenthesis.

^c^Clinically relevant oseltamivir-resistance molecular marker.

^d^Principal molecular marker of baloxavir-resistance. A cluster of seven viruses (based on collection date and strain name) containing PA-I38T was collected from a chicken farm in Arizona.

**Table S6.** Genomic analysis of M2 blocker-resistant viruses collected in Iowa.

| A(H5N1) virus | GISAID ID | Collection Date | Sequence differences | | | | | | | |
| --- | --- | --- | --- | --- | --- | --- | --- | --- | --- | --- |
|  |  |  | M2 | | PB2 | | PA | | NA | |
|  |  |  | Aa | Nt | Aa | Nt | Aa | Nt | Aa | Nt |
| Poultry |  |  |  |  |  |  |  |  |  |  |
| A/chicken/Iowa/24-037489-001/2024 | EPI_ISL_19711062 | 12-12-2024 | **S31N** | . | . | . | . | . | . | . |
| A/chicken/Iowa/24-037489-002/2024 | EPI_ISL_19711063 | 12-12-2024 | **S31N** | . | . | . | . | . | . | . |
| A/chicken/Iowa/24-037489-003/2024 | EPI_ISL_19711064 | 12-12-2024 | **S31N** | . | . | . | . | . | . | . |
| A/chicken/Iowa/24-037490-001/2024 | EPI_ISL_19711065 | 12-13-2024 | **S31N** | . | . | . | . | . | . | . |
| A/chicken/Iowa/24-037490-002/2024 | EPI_ISL_19711066 | 12-13-2024 | **S31N** | . | . | . | . | . | . | . |
| Human |  |  |  |  |  |  |  |  |  |  |
| A/Iowa/124/2024 | EPI_ISL_19669941 | 12-17-2024 | **S31N** | . | . | A207G | C489S | T1465A | I8T/I, V53I/V | C23T/C, G157A/G |

HPAI A(H5N1) viruses from poultry and a human case shown were collected from O’Brien County, Iowa. Other segments (not shown) are 100% identical in sequence at the nucleotide (Nt) and amino acid (Aa) levels. M2 substitution in **bold** indicates M2 blocker-resistance marker.

**Table S7.** Enzyme activity of recNA A(H5N1) proteins using large natural substrate fetuin in enzyme-linked lectin assay.

| **NA mutation** | **N2 numbering** | **Relative NA activity (%)** |  |
| --- | --- | --- | --- |
|  |  |  |  |
| **A/CA/148/2024 (genotype B3.13)** | |  |  |
| WT | - | 100 |  |
| H**275**Y | 274 | 76 |  |
| **A/WA/239/2024 (genotype D1.1)** | | |  |
| WT | - | 100 |  |
| H275Y | 274 | 127 |  |

Data are mean from four results.

**References**

[1] Rolfes MA, Kniss K, Kirby MK, et al. Human infections with highly pathogenic avian influenza A(H5N1) viruses in the United States from March 2024 to May 2025. Nat Med. 2025.

[2] Gao R, Pascua PNQ, Chesnokov A, et al. Antiviral Susceptibility of Swine-Origin Influenza A Viruses Isolated from Humans, United States. Emerg Infect Dis. 2024;30(11):2303-2312.

[3] WHO, 2024. Laboratory methodologies for testing the antiviral susceptibility of influenza viruses: Neuraminidase inhibitor (NAI). [cited 2025 Septemeber 10]. Available from: <https://www.who.int/teams/global-influenza-programme/laboratory-network/quality-assurance/antiviral-susceptibility-influenza/neuraminidase-inhibitor>

[4] Nguyen HT, Chesnokov A, De La Cruz J, et al. Antiviral susceptibility of clade 2.3.4.4b highly pathogenic avian influenza A(H5N1) viruses isolated from birds and mammals in the United States, 2022. Antiviral Res. 2023;217:105679.

[5] Gao R, Pascua PNQ, Chesnokov A, et al. Antiviral Susceptibility of Swine-Origin Influenza A Viruses Isolated from Humans, United States. Emerg Infect Dis. 2024;30(11):2303-2312.

[6] Colman PM, Varghese JN, Laver WG. Structure of the catalytic and antigenic sites in influenza virus neuraminidase. Nature. 1983;303(5912):41-4.

[7] Andreev K, Jones JC, Seiler P, et al. Antiviral Susceptibility of Highly Pathogenic Avian Influenza A(H5N1) Viruses Circulating Globally in 2022-2023. J Infect Dis. 2024;229(6):1830-1835.

[8] WHO, 2024. Laboratory methodologies for testing the antiviral susceptibility of influenza viruses: Polymerase acidic (PA) inhibitor, Baloxavir. [cited 2025 September 10]. Available from: <https://www.who.int/teams/global-influenza-programme/laboratory-network/quality-assurance/antiviral-susceptibility-influenza/polymerase-acidic-protein-inhibitor>

[9] Patel MC, Flanigan D, Feng C, et al. An optimized cell-based assay to assess influenza virus replication by measuring neuraminidase activity and its applications for virological surveillance. Antiviral Res. 2022;208:105457.
