# Supplementary figures and images for: Antiviral susceptibility of clade 2.3.4.4b highly pathogenic avian influenza A(H5N1) viruses from humans in the United States, October 2024 to February 2025
Source: Emerg Microbes Infect. 2025 Dec 15;15(1):2601372. doi: 10.1080/22221751.2025.2601372 (PMC12777753; doi:10.1080/22221751.2025.2601372)

Figure S1

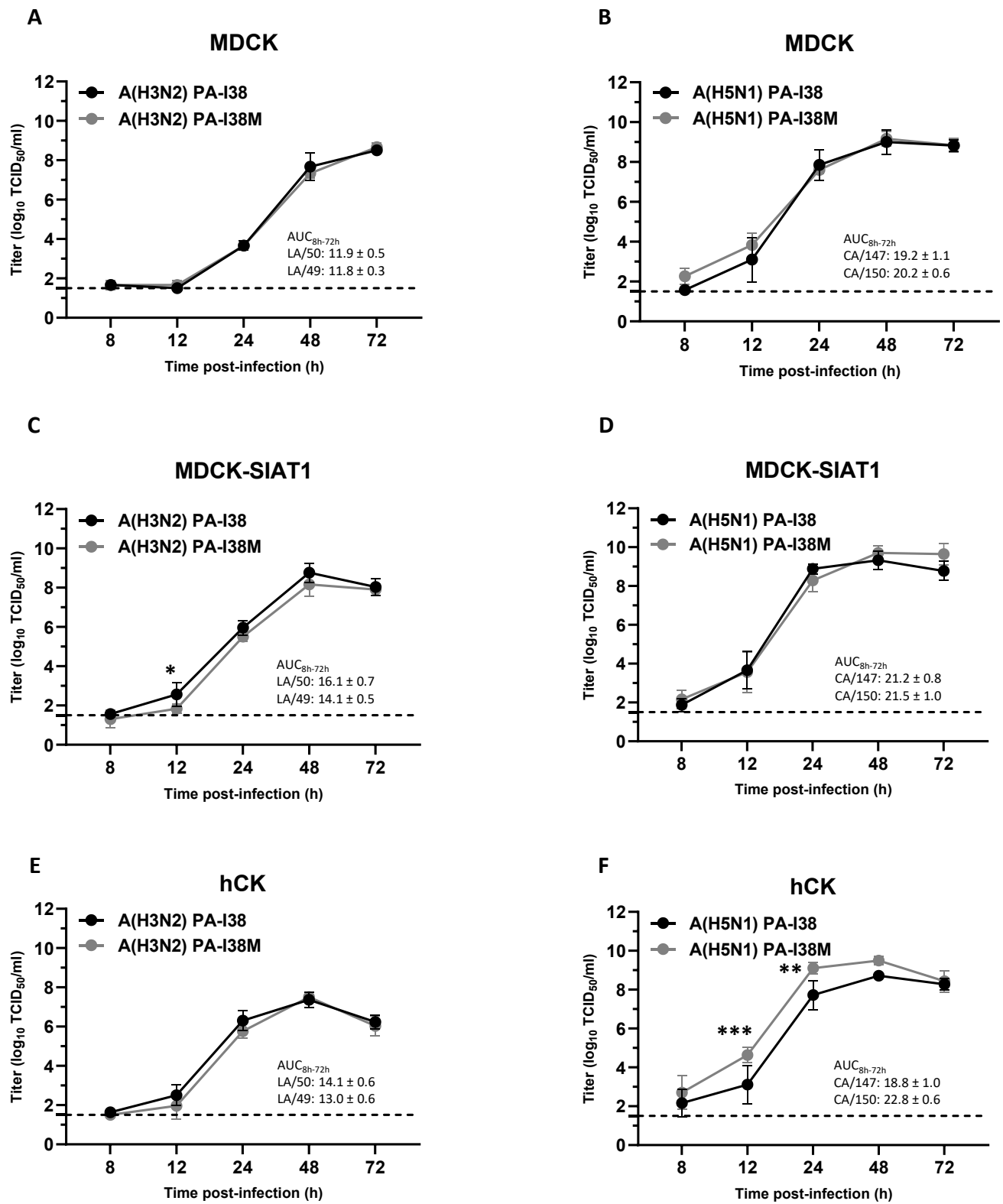

Figure S2.

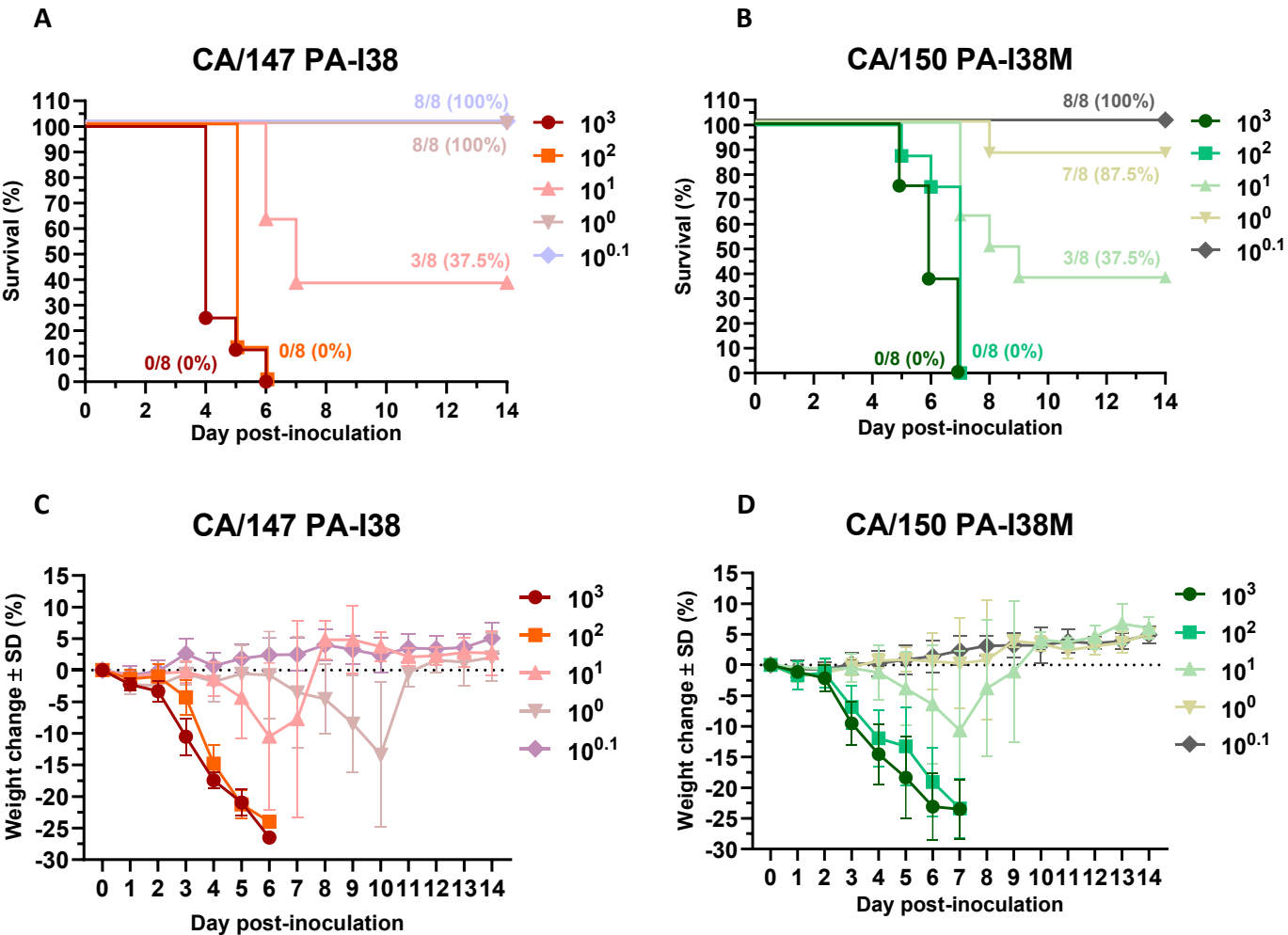

Supplement: Pascua_Update_H5_SuppFig_TEMi_2025_1441_R2.pdf [file TEMI_A_2601372_SM9200.pdf]
